# Preoperative optimization in hernia surgery: are we really helping or are we just stalling?

**DOI:** 10.1007/s10029-024-02962-9

**Published:** 2024-04-05

**Authors:** A. Fafaj, S. M. P. de Figueiredo, M. J. Rosen, C. C. Petro

**Affiliations:** Center for Abdominal Core Health, Digestive Disease and Surgery Institute, Cleveland Clinic Foundation, 9500 Euclid Avenue, Cleveland, OH 44195 USA

**Keywords:** Ventral hernia, Preoperative optimization, Prehabilitation

## Abstract

**Background:**

Managing patients with abdominal wall hernias and multiple comorbidities can be challenging because these patients are at increased risk for postoperative complications. Preoperative optimization has been used to identify and intervene upon modifiable risk factors to improve hernia repair outcomes, however, waiting to achieve optimization may cause unnecessary delays.

**Methods:**

We describe our approach to preoperative optimization in hernia and we review the current evidence for preoperative optimization.

**Conclusion:**

Modifying risk factors before undergoing elective hernia repair can improve the overall health of patients with multiple comorbidities. However, when considering the hernia-specific data, prolonging waiting times for patients to achieve full optimization is not justified. Surgeons should take a nuanced approach to balance achieving patient optimization without unnecessarily delaying surgical care.

## Introduction

Preoperative optimization has emerged as a strategy for intervening upon modifiable patient risk factors to improve outcomes. Optimizing patients aims to minimize the effect of some of these risk factors, can promote long-term health, and provides benefits beyond the perioperative period. However, it is important to recognize that effective prehabilitation programs can be challenging to implement requiring significant efforts from the surgeons and institutions. Importantly, while trying to achieve preoperative optimization, the surgeon should carefully balance holding patients accountable for improved health with hard arbitrary cut-offs and delaying surgical intervention. Striking the balance of achieving patient optimization without unnecessarily delaying surgical care is challenging and requires a nuanced approach. Ultimately, preoperative optimization should not be a “one size fits all” discipline. Here, we review the evidence behind preoperative optimization and present our approach when assessing these patients for hernia repair.

## Prehabilitation

Prehabilitation focuses on modifying patient risk factors to improve surgical outcomes. This is important in hernia patients because the hernia often limits their physical activities and often patient fear that any exercise will make the hernia worse thus leading to deconditioning. Some studies report possible benefits with prehabilitation; however, they are heterogenous in design and or lack statistical power. Liang et al. published one of the few trials evaluating the effect of prehabilitation on ventral hernia repair patients [1]. Patients with small to medium sized hernias (mean hernia area 38.2 cm^2^) were randomized to standard counseling or a prehabilitation program involving a nutritionist and physical therapist with weekly group meetings, daily checklist evaluating nutrition and exercise program including videos of different physical activities. Early results of this trial showed less wound complication in prehabilitation patients (6.8% vs. 17.6%, *p* = 0.167), though this did not reach statistical significance and those differences were driven mostly by surgical site occurrences (hematoma and seroma). The prehabilitation group was more likely to be hernia-free and complication-free (69.5% vs. 47.5%, *p* = 0.015) at 30 days [1]. However, at 2 years there were no differences in hernia-free and complication-free patients between the groups [2]. Another hernia-specific study by Renshaw et al. consisted of a multi-variable analysis of patients in the Abdominal Core Health Quality Collaborative (ACHQC) [3]. Higher patient-reported exercise level was associated with lower odds of postoperative complication and readmission when compared to patients who reported no physical activities. However, this was not a targeted intervention and might just represent patients at higher baseline activity have improved outcomes over less functional patients.

### How we do it

There is growing body of evidence supporting prehabilitation programs especially when done in a supervised setting. There is also emerging evidence in hernia patients showing potential for improved outcomes. It is important to highlight that prehabilitation requires a lot of resources and may not be feasible in all hernia clinics. In our practice, we recommend that all patients visit the ACHQC website (www.achsqc.org) which provides free resources for implementing an exercise program via mobile app which also includes an in-hospital guide for patients and physical therapists. The most critical part of any exercise programs is to take some time counseling patients in clinic that they can still perform exercise as tolerated without the fear of making their hernia larger. In the absence of any significant discomfort triggered by physical activity, the benefits of preoperative exercise likely outweigh any risk to potentiating the size or acuity of the hernia itself.

## Weight loss

By 2030, nearly half of US population will be obese with one in four expected to have a body mass index (BMI) > 35 kg/m^2^. With these alarming statistics we can expect an increase in the number of patients with obesity who present to our clinics with abdominal wall hernias. Surgeons are often reluctant to offer repair until an arbitrary BMI cutoff because there is an abundance of studies that link higher BMI with increase in hernia recurrence and wound morbidity. However, a careful review of these data brings into question the severity of the resultant wound morbidity and the consequences of these events. A National Surgical Quality Improvement Program (NSQIP) review of 25,172 patients who underwent ventral hernia repair, found that BMI > 30 kg/m^2^ was a significant predictor of postoperative surgical site infections (OR 1.49) [4]. Of note, open surgery was the strongest risk factor with OR 3.54. The pitfall with large studies from non-hernia-specific registries is that they lump hernia repairs by having two broad categories open vs. minimally invasive when we know that there is a wide variety of techniques within those categories. Some might involve raising large lipocutaneous flaps, while others can be performed without undermining the soft tissues. In addition, these large studies often produce statistically significant differences with minimal clinical importance. Hernia specific studies have shown how challenging it is to achieve meaningful weight loss. In a trial by Bernardi et al. looking at the benefits of a prehabilitation program, only two in ten patients were able to meet the 7% weight loss goal [2]. A separate prehabilitation study of 191 hernia patients showed a mean weight loss of 6 kg in patients participating in a weight loss program compared to 1.8 kg in patients who did not participate [5]. What is revealing in this study is that over half of patients in the weight loss program were lost to follow up and only one in ten patients met the weight loss goal and underwent hernia repair.

Another important consideration is the time required to achieve significant weight loss and the risk of a surgical emergency developing, which would result in worse outcomes and higher perioperative risk than elective cases [6]. In the trial by Bernardi et al., five patients (8.5%) randomized to prehabilitation underwent emergent hernia repair during the study period [2]. Schroeder et al. evaluated the use of preoperative bariatric surgery before ventral hernia repair in 12 patients and reported an average time to undergo hernia repair of 22.3 months, with 2 patients (16.7%) requiring emergent repairs [7]. Another study with 16 patients also evaluated preoperative bariatric surgery before ventral hernia repair, which occurred on average 22.6 months following the first visit to the abdominal wall surgeon. In this case series, two patients also required emergent repairs (12.5%) [8].

Given that patients with obesity are at increased risk for wound morbidity, minimally invasive techniques are preferred when feasible and expertise is available [9]. Addo et al. evaluated 151 patients with BMI > 35 kg/m^2^ undergoing MIS retromuscular repairs and found no differences in hernia recurrence or complications compared to patients with BMI < 35 kg/m^2^ [10]. Tsereteli et al. compared 134 patients with BMI > 40 kg/m^2^ undergoing laparoscopic intraperitoneal onlay mesh (IPOM) and found no differences in overall complications but did notice fewer recurrences in non-morbidly obese patients (8.3% vs. 2.9%) [11]. However, it is essential to highlight that patients in this study did not have the hernia defect closed, which likely impacted hernia recurrence rates, particularly in obese patients. A meta-analysis evaluating the effect of BMI in patients undergoing MIS ventral hernia repair, including 11 studies and 3199 patients, found similar hernia recurrence and complication rates using different BMI cut-offs [12].

### How we do it

We recognize the overall health benefits and the potential to improve outcomes in patients with obesity who present for hernia evaluation, and we encourage preoperative weight loss. A referral to our bariatric and metabolic clinic is always placed for patients who are willing to be evaluated for bariatric surgery or being placed on medical weight loss therapy. At the same time, we acknowledge how difficult it is to achieve weight loss and no longer use a hard BMI cutoff for offering surgery. Instead, we use an individualized approach when treating these complex cohort. When possible, we offer MIS repair since this technique has been shown to have fewer wound events. If an open technique is required, we bring patients back to clinic after the weight management referral to assess the progress every 3 months. If a patient has significant symptoms or weight loss goals have not been met despite sincere patient efforts, we offer repair after discussing the risk and benefits of surgery with the patients. Additionally, we utilize the thickness of the abdominal wall subcutaneous fatty tissue in the area of the incision to guide our decision to operate over an arbitrary BMI cutoff.

## Diabetes

Several studies in different types of surgeries have shown the negative impact of poorly controlled diabetes mellitus (DM). These results are often extrapolated to include hernia patients, so the consensus has been to avoid elective hernia repair in patients with HbA1c > 8%. However, despite being one of the most challenging public health issue, DM is poorly studied in hernia patients. The European Hernia Society conducted a systematic review to evaluate patient prehabilitation prior to ventral hernia repair. Only five publications discussed DM and were included for analysis [13]. Four were retrospective reviews that reported increased risk for surgical site occurrences and hernia recurrence in diabetic patients. The remaining study was an expert consensus statement based on systematic reviews [14]. Importantly, the data gathered were not from hernia-specific studies and showed increased odds of 1.69–5.8 for postoperative complications when HBA1C was > 6.0–7.0%. A more recent retrospective review of prospectively collected data on the hernia-specific Abdominal Core Health Quality Collaborative (ACHQC) database looked at the association of HbA1c and outcomes of ventral hernia repairs in diabetic patients [15]. The patients were divided into two groups (HbA1c < 8% and HbA1c ≥ 8%). After analyzing over two thousand patients, this study found no clinically significant differences in the rates of wound complications, reoperations, readmissions, length of stay or mortality.

### How we do it

We check HbA1C for all patients with DM before surgery. For those with HbA1c > 8%, we determine whether it is due to poor compliance or lack of medical access. For those with poorly controlled diabetes, a referral to endocrinology is made while emphasizing the importance of glycemic control on the patient’s overall health. Currently, we do not delay hernia repair until the HbA1c is lowered to some arbitrary cutoff. Finally, DM can be associated with morbid obesity, so we place a referral to either medical or surgical weight loss prior to elective hernia repair if the patient is willing to participate in these programs. Again, a nuanced approach should consider the diligence of the patient’s effort and hernia symptom severity.

## Smoking

Our evolution over the years with how we approach smoking patients highlights some of the pitfalls that exist in hernia surgery literature. Despite growing evidence that smoking is a leading cause of death, around 33% of adult males and 7% of adult females are active smoker worldwide [16]. Studies highlighting the negative effects of smoking are ubiquitous in several specialties such as urology, orthopedic and gastrointestinal surgery [17–19]. In hernia surgery, several large registry studies have demonstrated increased risk for wound complications. DeLancey et al. analyzed 220,000 ventral and inguinal hernias in NSQIP and showed that smokers had increased risk for wound infection and dehiscence [20]. Importantly, the actual percentage differences of any wound complication were much smaller, 2.58% in current smokers and 1.69% in non-smokers. More recent publications have challenged this perceived increased risk in smokers undergoing hernia repair. An analysis of the Abdominal Core Health Quality Collaborative compared current smokers with never smokers and found similar rates of surgical site infection (4.1% vs. 4.1%, *p* = 0.98), surgical site occurrences requiring procedural intervention (SSOPI) (6.2% vs. 5.0%, *p* = 0.43), reoperation (1.9% vs. 1.2%, *p* = 0.39), and all 30-day morbidity (7.5% vs. 6.6%, *p* = 0.60). Smokers had higher rates of surgical site occurrences (12.0% vs. 7.4%, *p* = 0 0.03) which was driven by increased seromas [21]. Kudsi et al. published similar results when looking at clinical outcomes of robotic ventral hernia repair between smokers and non-smokers. Surgical site occurrences and infections were similar when comparing smokers to nonsmokers (7.6% vs. 5.4%, *p* = 0.472; 5 vs. 0, *p* = 0.060, respectively) [22]. Based on these data our group made dramatic shift and no longer mandate smoking cessation prior to abdominal wall reconstruction. We then reviewed the data after the practice change by comparing 106 current smokers with a well-matched group of 304 never smokers. At 30 days, the rates of SSI (12.2% vs. 6.9%, *p* = 0.13), SSO (21.7% vs. 13.2%, *p* = 0.052), SSOPI (11.3% vs. 6.3%, *p* = 0.13) were not statistically significant between smokers and never smokers, respectively [23]. Notably, the smoking group showed a trend of higher wound morbidity and had a higher incidence of pneumonia (5.7% vs. 0.7%, *p* = 0.005).

### How we do it

Historically, we viewed smoking as a contraindication to hernia repair. Elective hernia repair was not scheduled until the patient reported absolute smoking cessation which was confirmed with a urine test. While it is difficult to ignore the large body of evidence implicating smoking in postoperative complications, we realized that the most cited large data pools lack the granularity of hernia-specific techniques and often produce statistically significant results that have triggered disingenuous conversations with patients regarding the true risk of active smoking in this context. Currently, we provide preoperative counseling and provide the appropriate referrals to those who are motivated to pursue smoking cessation because we recognize the overall health benefits. However, we are truthful when presenting data to smokers who present for hernia evaluation. We proceed with surgery even if the patient fails to quit smoking understanding there is an increase in the surgical site occurrence rate, the majority of which does not require procedural intervention and pulmonary complications. As with any significant practice changes, it is critically important to track outcomes. We are continuously doing so and our data from reviewing our outcomes on smokers is used as part of the shared decision making with active smokers who presented with hernias.

## Preoperative adjuncts

One of the key goals for treatment of ventral hernia is to achieve complete closure of the midline. Incomplete closure has been associated with increased wound morbidity and recurrence rates. Even with advancements in component separation techniques, complex hernias with loss of domain often have chronically retracted muscles reducing the compliance of the abdominal wall which impedes fascial closure. To combat this, preoperative adjuncts have been developed to aid with intraoperative fascial closure. The three most commonly used adjuncts are botulinum toxin A (BTA), progressive preoperative pneumoperitoneum (PPP), and soft tissue expanders.

Botulinum toxin A is a neurotoxin produced by bacterium *Clostridium botulinum* and it is typically injected under ultrasound guidance into the lateral abdominal wall muscles. The result is muscle paralysis causing muscle elongation and relaxation which is thought to increase abdominal compliance and fascial closure rates. A retrospective propensity-scored matched study by Deerenberg et al. reported on patients who underwent abdominal wall reconstruction with BTA (75 patients) and without BTA (145 patients). Patients with BTA had higher fascial closure rates (92% vs. 81%, *p* = 0.036) [24]. A subsequent study by the same group showed no difference in fascial closure rates when BTA and preperitoneal mesh placement were compared with component separation alone (100% vs. 90.5%; *p* = 0.11, respectively) [25]. A different propensity score matched study looked at the ACHQC registry and compared the fascial closure rate with and without BTA use. There was no difference in fascial closure rates between treatments with and without BTA (86% vs. 85.2% *p* = 0.934, respectively) [26].

Preoperative progressive pneumoperitoneum, first used in 1947, involves using an intraperitoneal catheter to fill the intraabdominal cavity with gas, air is commonly used. The volume of gas is incrementally increased until a predetermined volume of gas, or until the patient can no longer tolerate the procedure. The gas is thought to act as a pneumatic soft tissue expander allowing for elongation of soft tissues, improvement in respiratory capacity and tension free abdominal closure. High quality data for this technique is lacking. A meta-analysis with 1216 patient pooled the data from 53 studies and showed an 86% fascial closure rate when PPP was used, however, there was no comparison arm [27]. Importantly, there was a 12% rate of complications. Most were minor (abdominal pain, dyspnea and subcutaneous emphysema), however, there were five deaths related to PPP.

Soft tissue expanders are implants placed in various position in the abdominal wall such as intramuscular, between muscle layers, subcutaneously with the goal to mobilize soft tissue and fascia to facilitate reconstruction. The implants are typically left in situ until there is adequate soft tissue recruitment. The data for this adjunct are extremely limited consisting of mainly case series with no comparative arm.

### How we do it

We do not use BTA because there are no high-quality objective data to suggest that this adjunct facilitates fascial closure. When looking at the ACHQC data over 85% of cases had complete fascial closure without BTA. The challenge remains preoperatively identifying the 15% of patient who did not get complete fascial closure and randomizing them to either receive BTA or placebo to see the true of BTA on fascial closure. In addition, the use of BTA is off label when injected into the lateral abdominal wall muscles and it adds significant cost to the procedure so ideally it should only be used in trial settings. Although, we do not use PPP or soft tissue expanders routinely, we believe that there may be selects cases which could benefit from these techniques.

## Conclusion

Most of the hernias that the reconstructive surgeons evaluate qualify for elective repair, so we have the option to maximally optimize every modifiable risk factor to improve surgical outcomes. While there is no question that modifying these risk factors can improve the patient’s overall health, the hernia-specific data simply does not justify prolonged waiting times or even denying surgery when patients fail to achieve full optimization. With patients continuing to suffer while in the “waiting period”, one wonders whether we are truly helping or perhaps disserving them by continuously kicking the can down the road.

## References

[1] Liang MK, Bernardi K, Holihan JL, Cherla DV, Escamilla R, Lew DF (2018). Modifying risks in ventral hernia patients with prehabilitation: a randomized controlled trial. Ann Surg.

[2] Bernardi K, Olavarria OA, Dhanani NH, Lyons N, Holihan JL, Cherla DV (2022). Two-year outcomes of prehabilitation among obese patients with ventral hernias: a randomized controlled trial (NCT02365194). Ann Surg.

[3] Renshaw SM, Poulose BK, Gupta A, Di Stasi S, Chaudhari A, Collins C (2021). Preoperative exercise and outcomes after ventral hernia repair: making the case for prehabilitation in ventral hernia patients. Surgery.

[4] Kaoutzanis C, Leichtle SW, Mouawad NJ, Welch KB, Lampman RM, Wahl WL (2015). Risk factors for postoperative wound infections and prolonged hospitalization after ventral/incisional hernia repair. Hernia.

[5] Maskal SM, Boyd-Tressler AM, Heinberg LJ, Montelione KC, Petro CC, Krpata DM (2022). Can a free weight management program “move the needle” for obese patients preparing for hernia surgery?: outcomes of a novel pilot program. Hernia.

[6] Simon KL, Frelich MJ, Gould JC, Zhao HS, Szabo A, Goldblatt MI (2015). Inpatient outcomes after elective versus nonelective ventral hernia repair. J Surg Res.

[7] Schroeder AD, Mukherjee T, Tashjian N, Siu M, Fitzgibbons R, Nandipati K (2021). Staged complex abdominal wall hernia repair in morbidly obese patients. Hernia.

[8] Morrell DJ, Pauli EM, Lyn-Sue JR, Haluck RS, Rogers AM (2021). Laparoscopic sleeve gastrectomy in patients with complex abdominal wall hernias. Surg Endosc.

[9] Bittner R, Bain K, Bansal VK, Berrevoet F, Bingener-Casey J, Chen D (2019). Update of Guidelines for laparoscopic treatment of ventral and incisional abdominal wall hernias (International Endohernia Society (IEHS))—Part A. Surg Endosc.

[10] Addo A, Lu R, Broda A, George P, Huerta N, Park A (2021). Impact of Body Mass Index (BMI) on perioperative outcomes following minimally invasive retromuscular abdominal wall reconstruction: a comparative analysis. Surg Endosc.

[11] Tsereteli Z, Pryor BA, Heniford BT, Park A, Voeller G, Ramshaw BJ (2008). Laparoscopic ventral hernia repair (LVHR) in morbidly obese patients. Hernia.

[12] De Figueiredo SMP, Mao RMD, DelaTejera G, Tastaldi L, Villasante-Tezanos A, Lu R (2023). Body Mass Index Effect on Minimally Invasive Ventral Hernia Repair: A Systematic Review and Meta-analysis. Surg Laparosc Endosc Percutan Tech.

[13] Jensen KK, East B, Jisova B, Cano ML, Cavallaro G, Jørgensen LN (2022). The European Hernia Society Prehabilitation Project: a systematic review of patient prehabilitation prior to ventral hernia surgery. Hernia.

[14] Liang MK, Holihan JL, Itani K, Alawadi ZM, Gonzalez JRF, Askenasy EP (2017). Ventral hernia management: expert consensus guided by systematic review. Ann Surg.

[15] Al-Mansour MR, Vargas M, Olson MA, Gupta A, Read TE, Algarra NN (2023). S-144 lack of association between glycated hemoglobin and adverse outcomes in diabetic patients undergoing ventral hernia repair: an ACHQC study. Surg Endosc.

[16] Dai X, Gakidou E, Lopez AD (2022). Evolution of the global smoking epidemic over the past half century: strengthening the evidence base for policy action. Tob Control.

[17] Tian R, Zheng F, Zhao W, Zhang Y, Yuan J, Zhang B (2020). Prevalence and influencing factors of nonunion in patients with tibial fracture: systematic review and meta-analysis. J Orthop Surg Res.

[18] Tellini R, Mari A, Muto G, Cacciamani GE, Ferro M, Stangl-Kremser J (2021). Impact of smoking habit on perioperative morbidity in patients treated with radical cystectomy for urothelial bladder cancer: a systematic review and meta-analysis. Eur Urol Oncol.

[19] Brajcich BC, Yuce TK, Merkow RP, Bilimoria KY, McGee MF, Zhan T (2022). Association of preoperative smoking with complications following major gastrointestinal surgery. Am J Surg.

[20] DeLancey JO, Blay E, Hewitt DB, Engelhardt K, Bilimoria KY, Holl JL (2018). The effect of smoking on 30-day outcomes in elective hernia repair. The American Journal of Surgery.

[21] Petro CC, Haskins IN, Tastaldi L, Tu C, Krpata DM, Rosen MJ (2019). Does active smoking really matter before ventral hernia repair? An AHSQC analysis. Surgery.

[22] Kudsi OY, Kaoukabani G, Bou-Ayash N, Gokcal F (2023). Does smoking influence the clinical outcomes of robotic ventral hernia repair? A propensity score matching analysis study. J Robot Surg.

[23] Messer N, Melland MS, Miller BT, Krpata DM, Beffa LRA, Zheng X (2023). Evaluating the impact of lifting mandatory smoking cessation prior to elective abdominal wall reconstruction. A single-center experience. Am J Surg.

[24] Deerenberg EB, Shao JM, Elhage SA, Lopez R, Ayuso SA, Augenstein VA (2021). Preoperative botulinum toxin A injection in complex abdominal wall reconstruction—a propensity-scored matched study. Am J Surg.

[25] Marturano MN, Ayuso SA, Ku D, Raible R, Lopez R, Scarola GT (2023). Preoperative botulinum toxin A (BTA) injection versus component separation techniques (CST) in complex abdominal wall reconstruction (AWR): a propensity-scored matched study. Surgery.

[26] Horne CM, Augenstein V, Malcher F, Yunis J, Huang LC, Zolin SJ (2021). Understanding the benefits of botulinum toxin A: retrospective analysis of the Abdominal Core Health Quality Collaborative. Br J Surg.

[27] Martínez-Hoed J, Bonafe-Diana S, Bueno-Lledó J (2021). A systematic review of the use of progressive preoperative pneumoperitoneum since its inception. Hernia.

